# Effects of Liver Surgery on Drug Transporters in the Liver and Remote Organs

**DOI:** 10.1007/s11095-025-03901-8

**Published:** 2025-07-30

**Authors:** Reza Mehvar

**Affiliations:** Department of Biomedical and Pharmaceutical Sciences, School of Pharmacy, Chapman University, 9401 Jeronimo Road, Irvine, CA USA

**Keywords:** ischemia–reperfusion injury, liver surgery, liver transplantation, pharmacokinetics, transporters

## Abstract

Alterations in drug transporters in acute liver failure and chronic liver diseases, such as cirrhosis, have been reviewed before. However, there is a lack of comprehensive reviews on how liver surgery, including transplantation and partial hepatectomy, affects drug transporters. Because ischemia–reperfusion (IR) injury is a hallmark of liver transplantation and most other surgical procedures of the liver, this review focuses on the effects of IR injury, in addition to liver resection, on the expression and function of transporters in the liver and remote organs. Most of the reported studies in this area are carried out in animal models of liver surgeries, with relatively limited data in humans. The results indicate that the effects of IR injury and partial hepatectomy on drug transporters are complex and depend on many variables, such as the species, length and type of ischemia, reperfusion time, and the extent of liver resection. However, for a few major transporters, clear trends have emerged based on both animal and human studies. A major trend is that warm (normothermic) hepatic IR injury or liver transplantation causes overexpression of P-glycoprotein in the liver and remote organs, affecting the pharmacokinetics of substrate drugs. Another observed trend is the relocalization of the liver MRP2/Mrp2 from the canalicular membranes to the cytoplasmic area, reducing the function of the transporter even in the absence of a change in its protein. Alterations in transporter function, such as P-glycoprotein, may significantly impact the pharmacokinetics and pharmacodynamics of drugs in patients undergoing liver surgeries.

## Introduction

Liver transplantation and resection (partial hepatectomy) are surgical procedures required for various liver diseases. Liver transplantation is a life-saving treatment for multiple acute and chronic liver diseases, including alcohol-associated liver disease, non-alcoholic steatohepatitis, hepatitis C viral infection, cholestatic disease, hepatocellular carcinoma, and acute liver failure [1]. The procedure involves orthotopic replacement of the diseased organ with a liver procured from a deceased or living donor. The deceased donor organ has been traditionally obtained from brain-dead donors (i.e., donation after brain death or DBD) and has served as the main source of the donor pool. However, in recent years, livers from donors after cardiac death (i.e., donation after cardiac death or DCD) have also been included to expand the number of available organs for transplantation. Living donor liver transplantation (LDLT) is another procedure in which a portion of a living donor’s healthy liver is removed for transplantation into a recipient. LDLT offers patients on the organ transplant waiting list a path to liver transplantation regardless of their Model for End-Stage Liver Disease or MELD score [2].

Partial hepatectomy or liver resection is another liver surgery mainly used to remove benign lesions and primary or secondary malignancies of the liver. In addition, partial hepatectomy is conducted to procure the partial liver for LDLT. The liver has a remarkable capacity to regenerate rapidly after a partial hepatectomy or transplantation of the partial liver in LDLT [3, 4]. In rodents with 70% hepatectomy, the liver restores to its original size 7–10 days after the surgery, and in humans, the liver restores itself to its original size within 3 months after a right or left hepatectomy [4].

In addition to the medications unrelated to the liver surgery, patients undergoing liver surgery are usually required to take several medications after the operation, sometimes for life. Many of these drugs are subject to uptake or efflux transporters in the liver and other organs, which may affect their absorption, distribution, and elimination within the body and, ultimately, the pharmacodynamics of these drugs. For example, the pharmacokinetics and pharmacodynamics of calcineurin inhibitors (e.g., cyclosporine and tacrolimus), which are used as immunosuppressants following liver transplantation, may be affected by the activity of the drug efflux pump P-glycoprotein (P-gp). P-glycoprotein-mediated efflux may affect the blood and tissue concentrations of these drugs and their pharmacodynamics by reducing their absorption from the gastrointestinal tract [5–9] and distribution to peripheral blood mononuclear cells [10, 11]. Additionally, P-gp efflux reduces brain exposure [12, 13] and increases clearance of these drugs through P-gp-mediated drug excretion into the bile [13]. Mycophenolic acid (MFA), another immunosuppressant used in liver transplantation, is also a substrate for not only P-gp but also for the efflux transporter multidrug resistance-associated protein 2 (MRP2), affecting the oral absorption of the drug and excretion of the glucuronidated metabolites of the drug into the bile and urine [14, 15]. The glucuronidated metabolites of MFA are also substrates for OATP1B1 and OATP1B3 [15], which transport the MFA glucuronides from the blood to the liver, thus affecting the biliary excretion, enterohepatic recirculation, and the overall pharmacokinetics of MFA [16]. Therefore, potential changes in the expression or activity of these transporters due to liver surgery are expected to significantly affect the pharmacokinetics and pharmacodynamics of these drugs following the surgery.

Alterations in drug transporters in acute liver failure [17] and chronic liver diseases, such as cirrhosis, viral infections, and steatohepatitis [18, 19], have been reviewed before. Additionally, reviews of the pharmacokinetics of drugs in liver transplantation recipients have previously been published [20, 21]. However, a focused review of the effects of liver surgery, including liver transplantation and hepatectomy, on drug transporters appears to be lacking in the literature. Because ischemia–reperfusion (IR) injury is a hallmark of liver transplantation and most other surgical procedures of the liver, this manuscript will focus on the effects of IR injury, in addition to liver resection (hepatectomy), on the expression and function of transporters in the liver and remote organs. In addition to reviewing the effects of surgery on the expression of the drug transporters, the effects of these changes on the pharmacokinetics of drugs will be discussed whenever the data are available.

## Hepatic Ischemia–reperfusion Injury

Most liver surgeries include some form of blood flow interruption to the organ, which may result in IR injury. In particular, liver transplantation involves different types of IR injury during the liver procurement from the donor, transport to the recipient site, and implantation into the recipient [22]. Whereas DBD livers may not undergo substantial warm (normothermic) ischemia during the procurement process, DCD livers are usually subjected to some degree of warm ischemic periods because of the circulatory collapse in the recipient. After procurement, the liver is flushed with a cold preservation solution (like the University of Wisconsin solution) and kept at 4°C during storage or transport, resulting in cold ischemia. During transplantation into the recipient, the liver is again subjected to some period of warm ischemia before reperfusion is started in the recipient.

Cold ischemia occurs in the context of liver transplantation during liver storage and transport. Although cold ischemia reduces the liver's metabolic state, it still damages it due to oxygen shortage, lowering ATP levels, increasing cellular edema, and accumulating sodium [23–25]. Ironically, oxygenated blood flow after implantation stimulates the production of reactive oxygen species (ROS) and inflammatory cytokines, further harming the organ [23]. The damage from IR increases with the ischemic period. Prolonged preservation and the subsequent reperfusion cause hepatocellular injury, potentially leading to primary graft nonfunction and the need for retransplantation [24].

In contrast to cold ischemia, warm (normothermic) ischemia occurs both in the context of liver transplantation and other liver surgeries for hepatectomy or liver trauma. During hepatectomy or other liver surgeries, the liver may be subjected to the Pringle Maneuver, which consists of a complete stoppage of blood flow to the liver to avoid bleeding due to partial liver resection. Although warm and cold IR injuries share some common mechanisms, there are major differences in their pathophysiology, with warm IR injuries generally less tolerated, leading to quick cellular dysfunction and death [22, 26].

### Mechanisms of IR-induced Alterations in the Expression and/or Function of Transporters

The molecular mechanisms of hepatic IR injury are multifactorial and involve multiple liver cells and recruited neutrophils, leading to an excessive inflammatory response. The injury causes the generation or release of many inflammatory mediators, such as ROS, pro-inflammatory cytokines, damage‐associated molecular patterns (DAMPs), and adhesion molecules [27–29], which, in addition to compromising graft function, may alter the expression of liver transporters. Ischemia–reperfusion damage to hepatocytes and liver sinusoidal cells causes the release of DAMPs, such as high mobility group box 1 (HMGB1) and heat shock proteins, which activate Kupffer cells through toll-like receptors to release proinflammatory cytokines, such as TNF-α IL-1β, IFN-γ, and IL-12 [27]. Activation of Kupffer cells also causes the release of ROS [30], further damaging the liver. Both cytokines and ROS are known to affect the expression and function of both sinusoidal solute carrier (SLC) transporters and sinusoidal and canalicular efflux transporters in the liver. For example, it has been reported that whereas the exposure of hepatocytes to IL-1β or TNF-α usually reduces the mRNA levels of bile salt transporters and sinusoidal SLC transporters, the ATP-binding cassette (ABC) efflux transporters may be downregulated, upregulated, or remain unchanged by these cytokines [31–36]. Further, ROS causes overexpression of mdr1b mRNA and P-gp in primary rat hepatocytes and hepatoma cell lines [37, 38].

Additionally, liver ischemia causes a significant reduction in the ATP levels [39, 40], which are necessary for the function of ABC efflux transporters, such as P-gp and Mrp2, in the liver. Although the ATP levels somewhat recover during the reperfusion, they may remain below the baseline levels even 24 h after the reperfusion [41]. Lastly, liver IR injury is also reported to cause the relocation of the efflux transporters, such as Mrp2, from the canalicular membranes to the hepatocyte cytoplasm, thus affecting the Mrp2-mediated biliary excretion of drugs regardless of any potential changes in the transporter total protein content or ATP concentrations [42]. Collectively, all of these IR-induced molecular changes could alter the function of drug transporters in the liver in a complex and multidimensional manner, which would be hard to predict.

### Animal Models of Hepatic IR Injury

**Fig. 1:**
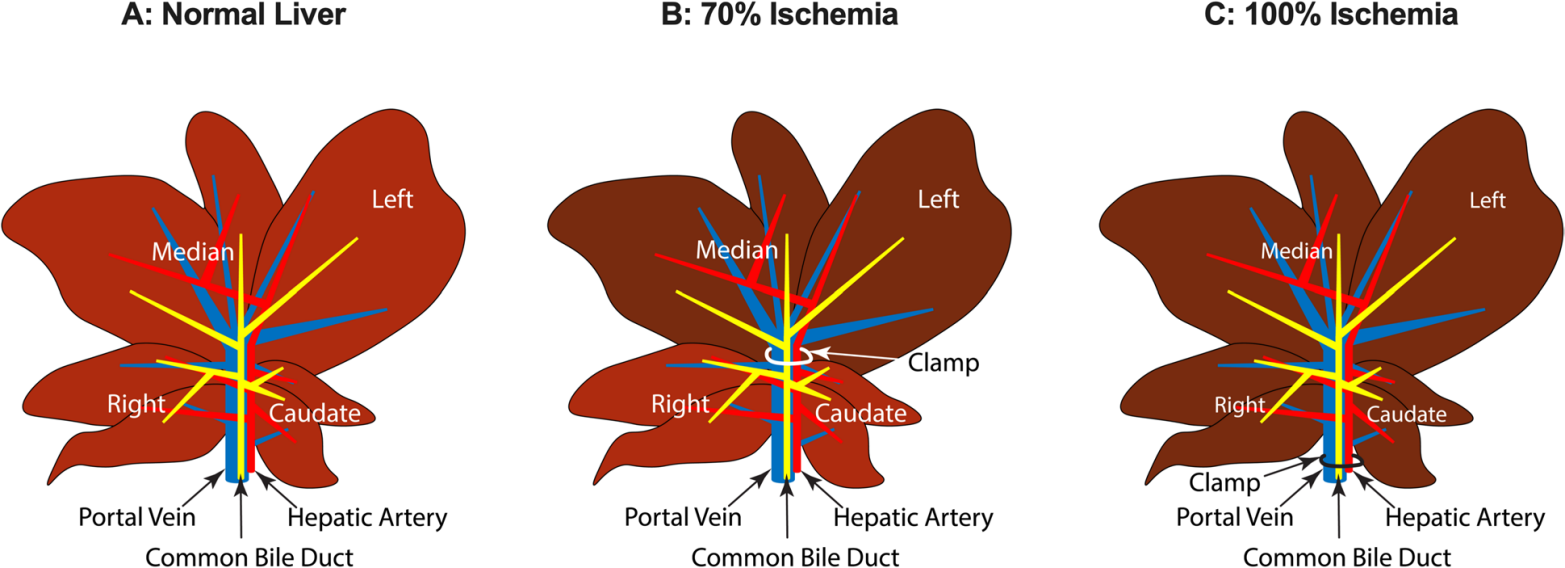
The liver lobes and portal vein, hepatic artery, and bile duct structures of normal liver (**A**) and 70% (**B**) and 100% (**C**) ischemia models in rats. In the 70% ischemia model (B), a vascular clamp is inserted on the portal triad above the caudate and right lobes to make the left and median lobes ischemic (darker color). In the 100% ischemia model (C), the clamp is inserted before the right and caudate lobe branches to make all the liver lobes ischemic.

As stated above, hepatic IR injury consists of warm (normothermic) and cold ischemia, depending on the type of surgery. Therefore, animal models of hepatic IR injury address these two types of injuries. Because of ease of handling, availability, and potential for genetic manipulations, rodent models have been extensively used in the studies of hepatic IR injury [43]. The rodent models use either partial (70%) or global (100%) ischemia (Fig. 1). The model of 70% partial ischemia is the most widely used experimental model of hepatic IR injury [43, 44]. This model is based on the interruption of blood supply (both portal vein and hepatic artery) and bile flow to the left lateral and median lobes of the liver for various periods (e.g., 30–120 min), leaving the blood supply to the right and caudate lobes intact (Fig. 1B) [44, 45]. Following the ischemic period, the blood flow is restored, and the IR injury is studied after different periods of reperfusion. This model allows studies of the effects of the time course of ischemia and/or reperfusion on the IR injury. The total interruption of the blood supply to the liver (100% ischemia, Fig. 1C), which is called the Pringle Maneuver, used in some human liver surgeries, is less common in rodents because the portosystemic collateral network in rodents is less developed than that in humans [46]. Therefore, global ischemic times longer than 30 min are potentially fatal in rodents unless there are additional procedures for portal decompression, such as portocaval shunts or spleen transposition [43, 46].

For the cold ischemia model, after procuring the liver from the animal model, the liver is stored at 4°C in a preservation fluid for various periods to simulate the storage of the donated livers and their transport for liver transplantation. The stored liver is then reperfused with a physiologic warm (normothermic) perfusate in an isolated liver perfusion model to simulate *in vivo* reperfusion in the recipient [47–51]. Similar to the warm IR injury model, the cold IR model allows for investigations of the effects of the time course of preservation and reperfusion on various liver parameters.

## Hepatic Drug Transporters

**Fig. 2:**
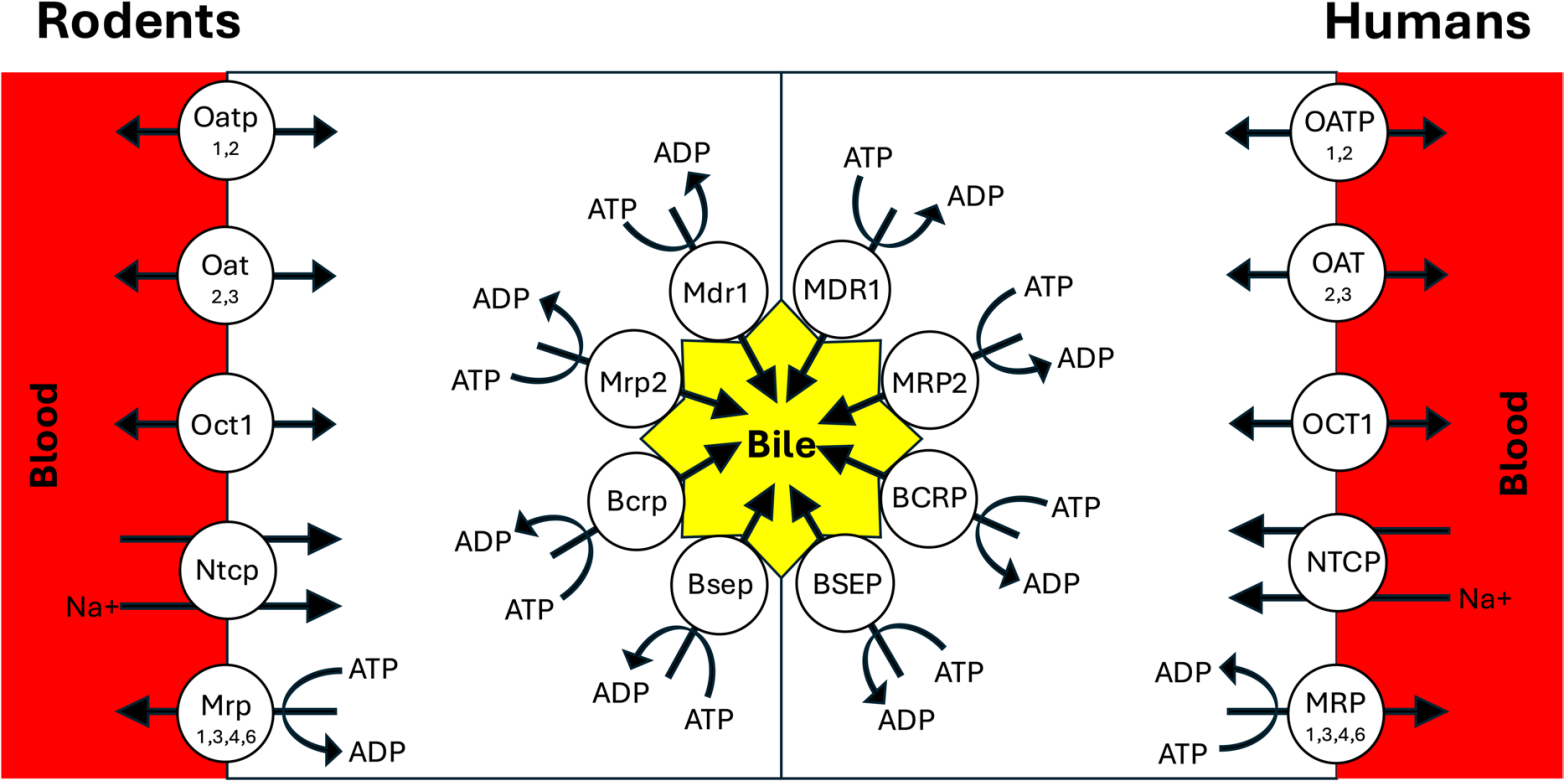
The major basolateral (blood-facing) and canalicular (bile-facing) drug transporters in the liver of humans and rodents.

The substrates for hepatic transporters are exogenous and/or endogenous. However, this review will focus on drug transporters, excluding those transporters with exclusively endogenous substrates. The hepatic drug transporters are generally characterized as influx and efflux transporters. Whereas the influx transporters carry drugs from the blood into the hepatocytes, the efflux transporters remove drugs from the hepatocytes by excreting them into the bile or the blood circulation (Fig. 2).

### Influx Transporters

Liver influx transporters, which are located on the basolateral membranes of hepatocytes (Fig. 2), are primarily part of the SLC transporters. These transporters are categorized as secondary active or facilitated transporters because they use ion gradients generated by the primary active transporters or electrochemical potential gradients, respectively, to transport drugs [52]. Although the SLC transporters are capable of bidirectional transport (Fig. 2), they usually transport their substrates from the blood to the hepatocytes and are thus called influx transporters. In the liver, the major SLC transporters include organic anion-transporting polypeptides (OATPs for humans and Oatps for other species), organic anion transporters (OATs for humans and Oats for other species), and organic cation transporters (OCTs for humans and Octs for other species) (Fig. 2).

Usually, OATPs/Oatps transport large hydrophobic organic anionic drugs and endogenous compounds. Bile acids, steroids, and thyroid hormones are examples of endogenous molecules, and statins, anti-cancer drugs, HIV protease inhibitors, angiotensin-converting enzyme inhibitors, and antibacterials are examples of drugs transported by the hepatic OATPs/Oatps [18, 52]. In the human liver, OATP1B1, OATP1B3, and OATP2B1 are the most abundant OATPs [53, 54] and play the most prominent role in the transport and pharmacokinetics of drugs [19]. In general, there is a broad substrate specificity for OATPs/Oatps, which show a significant degree of substrate overlap among their members [19].

OATs/Oats transport smaller, more hydrophilic organic anions into the hepatocytes, although neutral or even cationic molecules have also been reported to be substrates for these transporters. In humans and rats, OAT2/Oat2 and OAT3/Oat3 are expressed on the basolateral membranes of hepatocytes [52]. These transporters are responsible for the influx of several drugs, such as allopurinol, fluorouracil, methotrexate, salicylate, tetracycline, zidovudine, pravastatin, and warfarin, and endogenous molecules, such as bile acids, prostaglandins, and glutamate [18, 19, 52]. OAT7 is a more recently discovered member of these transporters, which is exclusively expressed in the liver and transports sulfate conjugates of endogenous steroids [55]. As opposed to other OATs, there are no known counterparts of OAT7 in mice or rats [54].

In the liver, OCTs/Octs usually transport small hydrophilic organic cations from the blood to the hepatocytes, although the transport of neutral or even anionic molecules has also been reported [56]. The major OCT/Oct expressed in the liver is OCT1/Oct1 (Fig. 2). Whereas human OCT1 is mainly expressed in the liver, rodent Oct1 is highly expressed in the liver, intestine, and kidney [52, 57]. OCT1/Oct1 transports endogenous compounds such as serotonin, dopamine, and norepinephrine and several drugs and vitamins, such as thiamine, quinidine, quinine, acyclovir, ganciclovir, morphine, paclitaxel, and metformin [52, 57].

In addition to SLC transporters, sodium-taurocholate co-transporting polypeptide (NTCP/Ntcp) is exclusively expressed on the basolateral membranes of hepatocytes (Fig. 2) and mediates the one-way sodium-dependent transport of bile acids from the blood to the hepatocytes. In addition to bile salt transport, NTCP/Ntcp also transports endogenous molecules, like estrone-3-sulfate, and drugs, like statins, to the hepatocytes [19, 31].

### Efflux Transporters

The hepatic efflux transporters are located either on the basolateral side, extruding drugs from the hepatocyte to the blood, or on the canalicular side, excreting drugs from the hepatocytes to the bile (Fig. 2). Both types of transporters result in a lower concentration of their substrates in the hepatocytes. The drug efflux transporters are part of the superfamily of the ABC transporters, which actively transport drugs using the energy obtained from converting ATP to ADP (Fig. 2).

The major efflux transporters on the hepatic basolateral membrane are the multidrug resistance-associated protein family (MRPs/Mrps). Whereas MRP2/Mrp2 is expressed on the canalicular side (discussed below), other MRPs/Mrps are located on the hepatic basolateral membranes (Fig. 2). The basolateral MRPs/Mrps actively transport a variety of organic anions, including endogenous compounds and drugs and their conjugated metabolites from the hepatocytes to the blood. They also transport bile acids and their conjugates into the blood [18, 58, 59].

The major canalicular drug efflux transporters are depicted in Fig. 2. Multidrug resistance protein 1 (MDR1/Mdr1), which is known as P-glycoprotein (P-gp), has been most extensively studied in the context of resistance to anticancer drugs. In the liver canalicular membranes, P-gp is very abundant and excretes many drugs, including immunosuppressants, cardiac drugs, HIV protease inhibitors, and anticancer drugs, into the bile [58]. The substrates of P-gp belong to diverse groups of chemical structures, and aside from the amphipathic nature of these drugs, there are few common structural features of the P-gp substrates. Additionally, P-gp expression and function exhibit a high degree of polymorphism [60].

As mentioned above, MRP2/Mrp2 is the only member of the MRP/Mrp family that is expressed on the hepatocyte canalicular membranes (Fig. 2). Substrates transported by MRP2/Mrp2 are both endogenous, like bilirubin and its glucuronidated conjugates and bile acids, and exogenous, like anticancer drugs and HIV protease inhibitors [58]. Additionally, this transporter substantially contributes to the bile acid-independent bile flow through active secretion of reduced glutathione into the bile [61].

Breast cancer resistance protein (BCRP/Bcrp) is another ABC efflux transporter, which is prominently expressed on the hepatocyte canalicular membranes (Fig. 2). A broad range of compounds, including organic ions, sulfate conjugates, and both positively and negatively charged molecules, are substrates for this transporter [18]. These substrates include anticancer drugs, such as anthracenes, camptothecin derivatives, and polyglutamates, nucleoside analogs, such as zidovudine and lamivudine, and many other classes of drugs [58]. Endogenous substances, like estrone 3-sulfate and dehydroepiandrosterone sulfate, are also substrates for this transporter. There is some overlap between substrates of BCRP/Bcrp and those of P-gp and MRP2/Mrp2 [54].

Bile salt export pump (BSEP/Bsep) is a canalicular efflux transporter (Fig. 2) whose main function is the efflux of conjugated and unconjugated bile acids from the hepatocytes into the bile [54]. However, in addition to bile salts, BESP/Bsep also excretes a few drugs, like pravastatin [62] and fexofenadine [63], into the bile.

## Effects of Hepatic Surgery on Drug Transporters

Most investigations regarding the effects of liver surgery on the hepatic drug transporters are conducted in animals, particularly rodents.

### Effects of Hepatic IR Injury on Hepatic Drug Transporters

Table I summarizes the literature on the effects of warm (normothermic) and/or cold hepatic ischemia on the liver’s basolateral and canalicular drug transporters. Interestingly, all of these studies are conducted in rats. The models used in these studies include warm partial (70%) or total (100%) IR, cold IR, a combination of warm and cold IR, cold IR followed by liver transplantation, or hepatic arterial ligation (Table I). As data in Table I indicate, the most frequently used model is warm partial (70%) hepatic IR, which shows no change or a reduction in the hepatic basolateral transporters. For example, reductions in the mRNA, proteins, or function of Ntcp [64, 65], Oatp [65, 66], and Mrp3 [67] have been reported in some models of warm 70% IR injury (Table I). In contrast, other studies [64, 68, 69] have shown no change in these basolateral transporters in this model. The differences among these studies may be related to the length of ischemia and/or perfusion. For example, a 24-h reperfusion model showed that the mRNA levels of Ntcp and Oatp1 remained unaltered after 60 min of ischemia [64]. However, subjecting the livers to 90 min of ischemia in the same study caused a decrease in their mRNA levels (Table I) [64].

**Table I:** Effects of Liver Ischemia–Reperfusion Injury on Drug Transporters in the Liver

Species	Intervention	Basolateral Transporter Change	Canalicular Transporter Change	Technique	Reference
*Warm Partial (70%) Ischemia/Reperfusion Models*					
SD Rats	*In vivo* warm partial (70%) ischemia (60 min)/reperfusion (24 h)	Ntcp⇔, Oatp1⇔	Bsep⇓, Mrp2⇔	mRNA/liver tissue	[64]
	*In vivo* warm partial (70%) ischemia (90 min)/reperfusion (24 h)	Ntcp⇓, Oatp1⇓	Bsep⇓, Mrp2⇓		
SD Rats	*In vivo* warm partial (70%) ischemia (90 min)/reperfusion (24 h)	Oatp1a4⇓	P-gp⇑, Mrp2⇓	WB/liver tissue	[66]
			P-gp⇓	Biliary clearance of RH-123	
			Mrp2⇓	Biliary clearance of RH-Glu	
		Oatp1a4⇓		Liver: plasma ratio of RH-123	
Wistar Rats	*In vivo* warm partial (70%) ischemia (60 min)/reperfusion (4 h and 24 h)	Mrp3⇓ at 4 h Mrp3⇔ at 24 h	Mrp2⇔ at 4 and 24 h	WB/liver tissue	[67]
		Oatp⇓ at 4 h^a^	Mrp2⇓ at 4 h^a^	fluorescein AUC in hepatocytes/Intravital multiphoton microscopy	
Wistar Rats	*In vivo* warm partial (70%) ischemia (60 min)/reperfusion (4 h)		P-gp⇓	Biliary excretion rate of Rh-123	[70]
SD Rats	*In vivo* warm partial (70%) ischemia (30 min)/reperfusion (1 h-7 days)	Ntcp⇔	Bsep/Mrp2⇔	WB/liver tissue	[68]
			Mrp2⇔	Ceftriaxone maximum transport capacity/biliary excretion	
SD Rats	*In vivo* warm partial (70%) ischemia (60 min)/reperfusion (0–48 h)	Ntcp⇓, Oatp1a1⇓, Oatp1a4⇓, Oatp1b2⇓, Mrp1⇔, Mrp3⇔, Mrp4⇔	Mdr1b⇑, Mrp2⇓, Bsep⇓	mRNA/liver tissue	[65]
SD Rats	*In vivo* warm partial (70%) ischemia (60 min)/reperfusion (0–48 h)	Mrp1⇔Mrp3⇔,Mrp4⇔	Mrp2⇓	mRNA and WB/liver tissue	[69]
Wistar Rats	*In vivo* warm partial (70%) ischemia (60 min/reperfusion (12 h)		P-gp⇑	WB/liver tissue	[71]
SD Rats	*In vivo* warm partial (70%) ischemia (60 min)/reperfusion (24 or 72 h)		P-gp⇓, Mrp2⇓ (24 h) P-gp⇔, Mrp2⇔ (72 h)	Biliary clearance in IPRL	[72]
Wistar Rats	*In vivo* warm partial (70%) ischemia (20 min)/reperfusion (60 min)		Mrp2 Internalization (from canalicular membrane to pericanalicular cytoplasm)	Immunofluorescence/liver tissue	[42]
*Warm Total (100%) Ischemia/Reperfusion Models*					
SD Rats	*In vivo* warm total (100%) ischemia (60 min)/reperfusion (60 min)		Mrp2⇓, Bcrp⇓	mRNA/liver tissue	[73]
SD Rats	*In vivo* warm total (100%) ischemia (20 min)/reperfusion (15 min or 8 h) with or without partial (70%) hepatectomy	Oatp1a4⇔	Mrp2⇑ (15 min reperfusion), Mrp2⇔ (8 h reperfusion)	WB/liver tissue	[74]
			Mrp2⇓ (15 min reperfusion), Mrp2⇔ (8 h reperfusion)	Fluorescein biliary excretion	
*Cold Ischemia/Reperfusion Models*					
Wistar Rats	Ex vivo cold (4°C) ischemia (8 h)/warm reperfusion (60 min)		Mrp2⇓	Biliary excretion of carboxyfluorescein/IPRL	[75]
			Mrp2 internalization (from canalicular membrane to cytoplasm)	Immunofluorescence/liver tissue	
SD Rats	Ex vivo cold (4°C) ischemia (24 h)/warm reperfusion (2 h)	Ntcp⇑, Oct1⇑, Oat2⇑, Oatp1a1⇔, Oatp1a4⇔, Oatp1b2⇔	Mdr1a⇓, Mrp2⇔, Bsep⇔, Bcrp⇔	mRNA/liver tissue	[76]
Wistar Rats	Ex vivo cold (4°C) ischemia (8–24 h)/warm reperfusion (60 min)		Mrp2⇔	WB/liver tissue	[77]
			Mrp2 internalization	Immuno-fluorescence/liver tissue	
			Mrp2⇓	Biliary excretion of carboxyfluorescein/IPRL	
Wistar Rats	Ex vivo cold (4°C) ischemia (44 h)/warm reperfusion (3 h)	Ntcp⇔, Oatp1a1⇔, Oatp1a4⇔, Oatp1b2⇔	Mrp2⇓, Bsep⇓	WB/liver tissue	[78]
		Ntcp⇓	Mrp2⇓,Bsep⇓	Immunofluorescence/hepatocytes & biliary excretion of taurocholate	
*Other Models*					
Wistar Rats	*In vivo* hepatic arterial ligation	Ntcp⇓ 24 h after surgery	Bsep⇓, Mrp2⇓ 24 h after surgery	mRNA/liver tissue	[79]
SD Rats	*In vivo* warm total (100%) ischemia (10–30 min) plus ex vivo cold (3 h) ischemia, followed by warm reperfusion (1 h)		Mrp2⇓	Fluorescein efflux/IPRL	[80]
SD Rats	Ex vivo cold (4°C) ischemia (1 h or 12 h), followed by liver transplantation (1, 3, 5, 7, 10, 14 days post-transplantation)	Ntcp: ⇓ (days 1–3), ⇔ (days 5–14) Oatp1: ⇓ (days 1–3), ⇔ (day 5), ⇑ (days 7–14) Oatp2 and Oatp4: ⇓ (day 1–7), ⇔ (day 14)	Bsep: ⇓ (day 1–3), ⇔ (day 5), ⇑ (days 7–14)	mRNA and WB^b^/liver tissue	[81]

^a^Delayed uptake and excretion of fluorescein was attributed to a decrease in the function of Oatp and/or Mrp2

^b^Except for Oatp1, WB changes were in agreement with the mRNA changes

Like the basolateral transporters, warm IR injury causes a reduction or no change in the mRNA or protein of Mrp2 and Bsep efflux transporters (Table I). In contrast, the mRNA or protein levels of P-gp are overexpressed (Table I) [65, 66, 71]. Tanaka *et al.* [65] showed that whereas the mRNA levels of basolateral influx transporters Ntcp, Oatp1a1, Oatp1a4, Oatp1b2, and canalicular efflux transporters Mrp2 and Bsep were reduced following warm partial (70%) hepatic IR injury, the mRNA levels of Mdr1b was increased 24 h after the injury in rats (Table I). This finding was confirmed in subsequent studies by us [66] and others [71], who showed warm IR injury increases the protein levels of P-gp in the liver (Table I). The warm IR-induced increase in the mRNA and protein levels of P-gp [65, 66, 71] is consistent with the previous reports indicating that warm IR injury is associated with significant increases in ROS [30] and that ROS causes overexpression of P-gp in primary rat hepatocytes and hepatoma cell lines [37, 38].

**Fig. 3:**
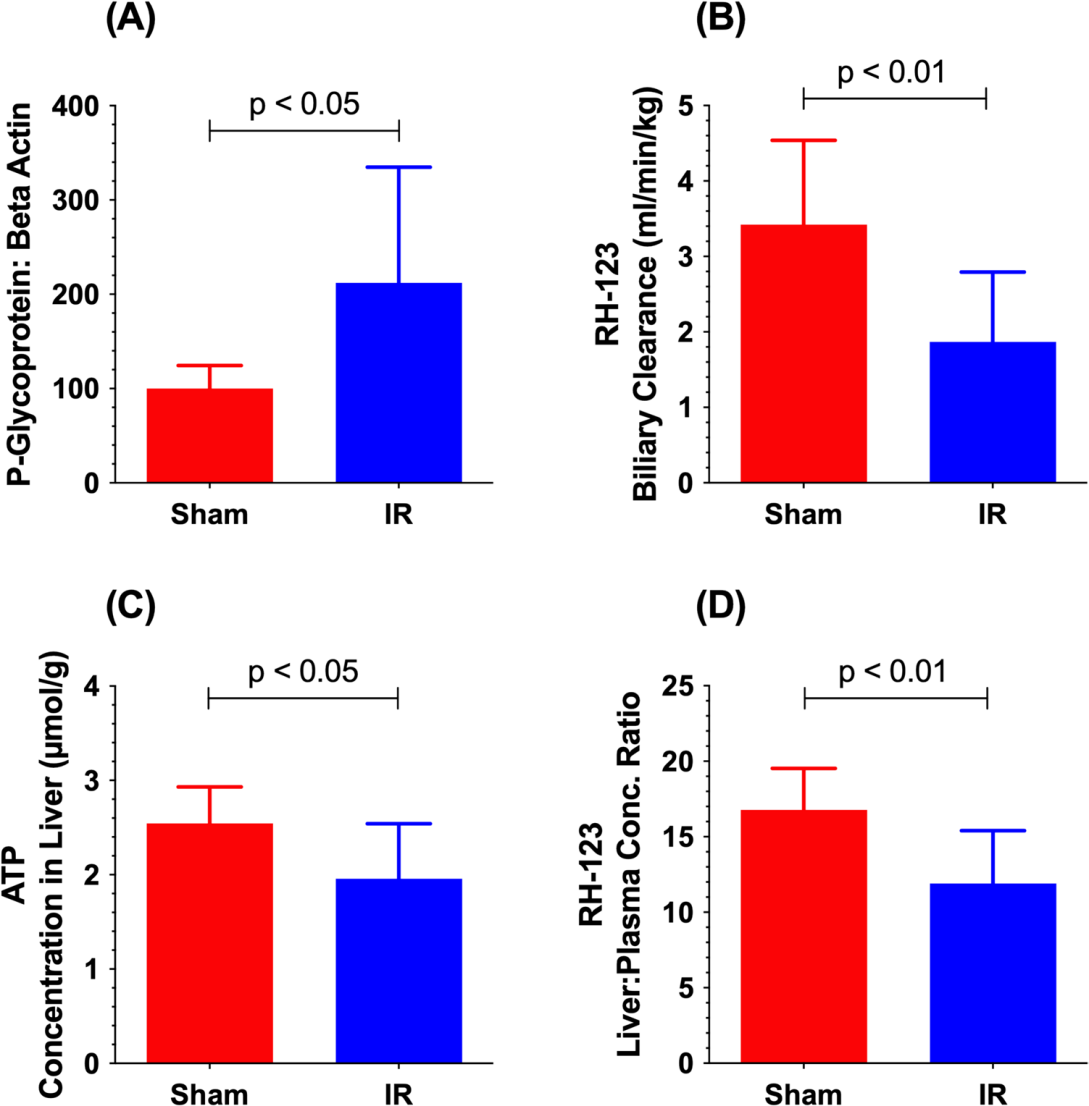
Terminal P-gp protein in the liver membrane fractions (**A**), *in vivo* biliary clearance of RH-123 (**B**), terminal liver ATP levels (**C**), and terminal liver: plasma concentration ratio of RH-123 (**D**) in rats subjected to sham surgery (Sham) or warm partial (70%) hepatic ischemia (90 min) and reperfusion (24 h) (IR) injury. Data, presented as mean (columns) and SD (error bars), are from Miah *et al.* [66].

Despite the warm IR injury-induced increases in the mRNA and protein levels of P-gp, we showed that biliary clearance of rhodamine 123 (RH-123), a P-gp substrate, is reduced in the livers subjected to the injury (Fig. 3) [66, 72]. A warm partial (70%) model of ischemia (90 min) and reperfusion (24 h) injury in rats resulted in a twofold increase in the P-gp protein in the liver membrane fractions (Fig. 3A) [66]. However, despite this increase, IR injury caused a 45% decline in the *in vivo* biliary clearance of RH-123 (Fig. 2B). Additional mechanistic studies revealed that IR injury also caused a significant reduction in the liver ATP levels 24 h after the reperfusion (Fig. 2C). Further, the liver: plasma concentration ratio of RH-123 was reduced because of IR injury (Fig. 2D), which was due to a reduction in the Oatp1a4 protein levels [66]. The influx transporter Oatp1a4 reportedly contributes to the uptake of RH-123 from the liver sinusoids to the hepatocytes [82]. Therefore, the reduction in the biliary clearance of RH-123 despite the higher protein levels of P-gp after the hepatic IR injury was attributed to both a reduction in the hepatic uptake of the P-gp marker and a lower ATP level due to IR injury [66].

As stated earlier, the model of total (100%) ischemia (i.e., Pringle maneuver) is less frequently used in rodents (Table I) because of limited portosystemic networks in rodents, resulting in significant mortality with ischemic times of more than 30 min. Using such a model with a 20 min warm ischemia time, we showed a significant increase in the protein levels of Mrp2 in the liver shortly (15 min) after the reperfusion, which returned to normal levels after 8 h of reperfusion (Table I) [74]. However, when the function of Mrp2 was tested using fluorescein (FL) and its glucuronidated metabolite, which are transported by Mrp2 from the hepatocytes into the bile, paradoxically, the biliary recovery and clearance of FL and the biliary recovery of FL glucuronide were drastically (70–80%) reduced after 15 min of reperfusion [74]. The reduction in the activity of Mrp2, despite the transient increase in its protein levels at 15 min after reperfusion, was associated with a significant decline in the bile flow rates and liver ATP levels [74]. Whereas the bile flow rates in the 15 min reperfusion group decreased by > 60%, they returned to control values at 8 h post-reperfusion [74]. Indeed, there were significant correlations between the bile flow rate and the biliary recovery of FL or FL-Glu with coefficients of determination (*r*^*2*^) of ≥ 0.9, suggesting that the decreases in the biliary recovery of FL and FL-Glu after 15 min of reperfusion despite an increase in the transporter protein levels were, likely, due to the decreased bile flow rates at this time point. In agreement with this postulate, 8 h after reperfusion, when bile flow rates were similar in the IR and sham animals, there was no change in the biliary recovery of FL or FL-Glu [74].

Similar to warm ischemia, the effects of cold IR injury on drug transporters also depend on the length of ischemia. However, in contrast to warm IR injury, which may cause the downregulation of basolateral transporters, cold IR injury may cause an upregulation of these transporters (Table I). In a rat model of 24 h cold ischemia, followed by 60 min of reperfusion in an isolated perfused liver model, Almazroo *et al.* [76] reported mRNA overexpression of Ntcp, Oct1, and Oat2. At the same time, no change was observed for Oatp1a1, Oatp1a4, or Oatp1b2. As for the efflux transporters, studies have shown that cold IR injury causes a redistribution of Mrp2 from the canalicular membranes to the cytoplasm, resulting in a reduction in the activity of this canalicular efflux transporter [75, 77]. In an elegant study, Kudo *et al.* [77] stored rat livers in cold (4°C) University of Wisconsin solution for 8 to 24 h, followed by reperfusion of the livers ex vivo. Cold ischemia caused a significant reduction in the bile output along with a delay in the biliary excretion of carboxyfluorescein, which is transported into the bile by Mrp2. However, Western blot analysis of the Mrp2 protein did not show any significant changes as a result of cold ischemia. Immunohistochemical analysis was then conducted to investigate the possibility of cold ischemia-induced hepatocellular relocalization of the transporter, which showed a significant reduction in the localization of Mrp2 in the bile canaliculi and an increase in the Mrp2 fluorescence in the cytoplasm [77]. The authors showed that the cold ischemia-induced internalization of Mrp2 is due to the activation of Kupffer cells because pretreating the rat livers with a Kupffer cell-depleting agent caused a redistribution of Mrp2 from the cytoplasm to the canalicular membranes. In addition to its potential role in graft dysfunction after transplantation by creating an imbalance in the generation and excretion of bilirubin and glutathione [77], the cold ischemia-induced relocalization of Mrp2 could significantly affect the biliary excretion of drugs following liver transplantation. The internalization of Mrp2 after cold ischemia has also been reported in an *in vivo* animal model of warm IR injury [42].

### Effects of Partial Hepatectomy on Hepatic Drug Transporters

Table II summarizes the studies investigating the effects of partial hepatectomy in rodents on the basolateral and canalicular membranes of the liver hepatocytes. As shown in the table, except for two studies in mice, all of the reports investigating the effects of partial hepatectomy on the hepatic transporters are conducted in rats (Table II). Additionally, a significant majority of the studies are related to two-thirds or ~ 70% partial hepatectomy, while there are a few studies after 90% hepatectomy (Table II).

**Table II:** Effects of Partial Hepatectomy on Drug Transporters in the Liver

Species	Intervention	Basolateral Transporter Change	Canalicular Transporter Change	Technique	Reference
SD Rats	Partial (70%) Hepatectomy		Mdr1a⇑, Mdr1b⇑ 24–48 h after surgery	mRNA/liver tissue	[83]
SD Rats	Partial (70%) Hepatectomy	Ntcp⇓ 24 h after surgery		mRNA and WB/liver tissue; Sodium-dependent taurocholate uptake in hepatocytes	[84]
SD Rats	Partial (70%) Hepatectomy	Ntcp⇓, Oatp1⇓, Oatp2⇓ 12–48 h after surgery, returned to normal 4–14 days after surgery	Mrp2/Bsep⇑ 12–48 h after surgery, returned to normal by 4–7 days after surgery	mRNA and WB/liver tissue	[85]
SD Rats	Partial (90%) Hepatectomy	Ntcp⇓ 1–3 days after surgery Mrp4⇑ 3 days after surgery	Bsep⇔	mRNA and WB/liver tissue	[86]
SD Rats	Partial (90%) Hepatectomy	Mrp3⇓ 1 day after surgery Mrp3⇑ 3 and 7 days after the surgery Oatp1⇓, Mrp1⇑ 1–3 days after surgery	Mrp2⇓ 1–2 days after surgery	mRNA/liver tissue	[87]
SD Rats	Partial (70%) Hepatectomy		Mrp2⇔36 and 72 h after hepatectomy	WB/liver tissue	[88]
	Partial (90%) Hepatectomy	Mrp3⇑ 36 and 72 h after hepatectomy	Mrp2⇓ 36 and 72 h after hepatectomy	WB/liver tissue	
Fischer Rats	Partial (70%) Hepatectomy		Mdr (P-gp)⇑ 24–72 h after surgery	mRNA/liver tissue	[89, 90]
Wistar Rats	Partial (70%) Hepatectomy	Ntcp⇓, Oatp1⇓, Oatp2⇓ 24 h after surgery	Mdr1a⇑, Mdr1b⇑, Mrp2⇓, Bsep⇓ 24 h after surgery	mRNA/liver tissue	[91]
		Ntcp⇓,Oatp2⇓, Oatp1⇑ 24 h after surgery	Mrp2⇔,Bsep⇔24 h after surgery	WB/liver tissue	
Wistar Rats	Partial (70%) Hepatectomy		P-gp⇓ at 12 h and ⇑ at 24 and 48 h after surgery	WB/Hepatocytes	[92]
Wistar Rats	Partial (70%) Hepatectomy		Mdr1b⇑ 24 h after surgery	mRNA/Hepatocytes	[93]
Wistar Rats	Partial (70%) Hepatectomy	Ntcp⇓, Oatp2⇓, Oatp3⇓, Oatp4⇓, Oatp5⇑, Oatp7⇓, Octn1⇑, Octn2⇑, Mrp6⇓ 3–48 h after surgery	BSEP⇔, Mrp2⇔ 3–48 h after surgery	mRNA/liver tissue	[94]
Wistar Rats	Partial (70%−75%) Hepatectomy		Mrp2⇔ 1 and 7 days after surgery	WB & Immunofluorescence/liver tissue	[95]
			Mrp2 ⇔ 1 day after surgery	Excretion Rate/Isolated Hepatocytes	
B6C3/F1 Mice	Partial (70%) Hepatectomy		Mdr1b⇔, Mdr1a⇑ 24–48 h after surgery	mRNA/liver tissue	[96]
C57Bl/6Mice	Partial (70%) Hepatectomy	Ntcp⇔, Oatp1a4⇑, Mrp3⇑, Mrp4⇔ 24 and 48 h after surgery Oatp1a1⇓, Oatp1b2⇓ 48 h after surgery	Mrp2⇑ 48 h after surgery Bsep⇔, Mdr1a⇔, Mdr1b⇔ 24 and 48 h after surgery	mRNA/liver tissue	[97]
		Ntcp⇔, Oatp1a1⇔, Mrp3⇑ 24 and 48 h after surgery Oatp1a4⇑ 24 h after surgery Oatp1b2⇑ 48 h after surgery	Bsep⇑, Mrp2⇔ 48 h after surgery	WB/liver tissue	

The studies in rats clearly and consistently show that partial hepatectomy significantly reduces the mRNA, protein, and function of the basolateral transporter Ntcp. For example, Green *et al.* [84] showed that 24 h after 70% partial hepatectomy in SD rats, the basolateral mRNA and protein expression of Ntcp was decreased by more than 95%. The decrease in the mRNA and protein expression of Ntcp was associated with a substantial reduction in the sodium-dependent taurocholate uptake by basolateral hepatocyte plasma membrane vesicles [84]. Other studies (Table II) [85, 86, 91, 94] also showed that the mRNA and protein levels of Ntcp are significantly reduced between 12 h and 3 days after both 70% and 90% partial hepatectomy in rats, which returned to normal values 4–14 days after the surgery [85]. In contrast to the multiple studies showing clear decreases in the mRNA, protein, and activity of Ntcp after partial hepatectomy in rats, one study in mice [97] showed that 70% hepatectomy did not alter the mRNA of Ntcp. The latter surprising finding was attributed to possible species differences in the regulation of Ntcp, such as the lack of FXR/PXRα response element in the mouse Ntcp promoter [97].

Another transporter that has reportedly been affected relatively consistently by partial hepatectomy in different studies in rats is the canalicular efflux transporter P-gp (Mdr1a/b) (Table II). Several studies [83, 89–93] have reported a significant overexpression of Mdr1a/b 24–72 h after 70% partial hepatectomy in rats, both at the mRNA and the protein (Western blot) levels (Table II). In one of the first reports on this subject, Thorgeirsson *et al.* [89] showed that the mRNA of the Mdr gene was elevated in the preneoplastic and neoplastic lesions of the rat liver. They also showed that the Mdr gene was significantly (over tenfold) overexpressed in regenerating rat livers 24–72 h after 70% partial hepatectomy, which returned to normal values at 120 h. A follow-up study [90] suggested that the reported hepatectomy-induced increased Mdr mRNA levels were due to posttranscriptional stabilization of the message rather than increased transcription. Teeter *et al.* [83] and Vos *et al.* [91] showed that among different Mdr genes, the hepatic Mdr1b mRNA was highly elevated. In contrast, the increase in the levels of Mdr1a was moderate after partial hepatectomy in rats. This observation in rats is different from that in mice, where the prominent increase was seen in the Mdr1a gene instead of the Mdr1b gene [96], suggesting a species-specific activation of the Mdr gene due to partial hepatectomy. In a later study, Daoudaki *et al.* [92] showed that the previously reported increase in the Mdr1b mRNA in the partially hepatectomized rats was also associated with increased protein levels of P-gp in these animals. These studies, which were conducted in hepatocytes isolated from the partially hepatectomized rats, showed that after an initial reduction at 12 h, the hepatocyte levels of P-gp were significantly increased at 24 and 48 h after the partial hepatectomy [92].

Changes to the influx and efflux transporters other than Ntcp and P-gp have also been reported after partial hepatectomy (Table II). However, the direction or extent of changes reported in different studies is not in complete agreement in some cases (Table II). These discrepancies may be related to methodological differences among these studies, including the time course of the observations.

### Effects of Hepatic IR or Partial Hepatectomy on Drug Transporters in Remote Organs

**Fig. 4:**
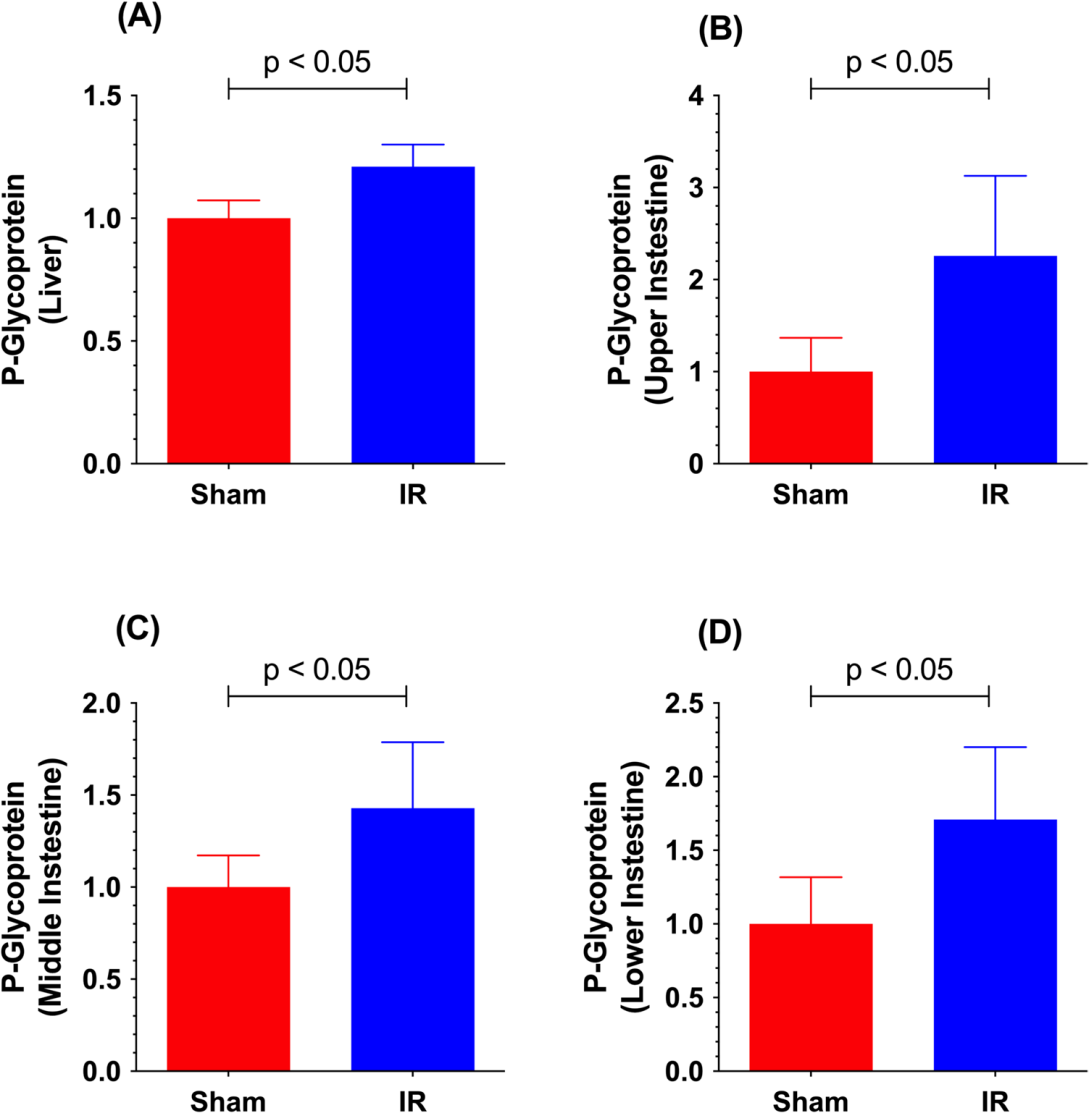
Terminal P-gp protein in the crude plasma membranes from the liver (**A**) and upper (**B**), middle (**C**), and lower (**D**) intestine of rats subjected to sham surgery (Sham) or warm partial (70%) hepatic ischemia (60 min) and reperfusion (12 h) (IR) injury. Data, presented as mean (columns) and SD (error bars), are from Ikemura *et al.* [71].

Table III summarizes studies on the effects of liver surgery on drug transporters in remote organs, such as the intestine, kidneys, and brain. In an elegant study, Okuda and colleagues [71] investigated the effects of 70% warm hepatic ischemia (60 min)-reperfusion (12 h) injury on the expression and function of intestinal P-gp in rats. In addition to a significant increase in the protein levels of P-gp in the liver, the liver IR injury caused a significant increase in the protein levels of P-gp in all sections (upper, middle, and lower) of the small intestine, with the upper intestine showing the largest (> twofold) increase (Fig. 4). The CYP3A level was also increased in the upper section of the small intestine but remained unchanged in the liver of rats subjected to IR injury. In agreement with these changes, the concentrations of cyclosporine, a substrate for both P-gp and CYP3A, in the portal blood were significantly decreased after administration of the drug into a loop containing the upper part of the intestine. In addition to the *in situ* absorption studies, *in vivo* experiments after oral administration of cyclosporine revealed a significant reduction in the blood concentrations and AUC of the drug in the IR animals after oral administration. However, there were no significant differences between the IR and sham animals in terms of cyclosporine AUC after the IV administration. Overall, these results suggest that the decreased oral availability of cyclosporine in the IR rats is likely due to a decrease in the oral absorption and/or increase in the first-pass metabolism of the drug in the intestine due to an increase in both P-gp and CYP3A in the upper parts of the intestine of IR rats. A subsequent study [98] by the same group showed that the administration of the antioxidant Trolox before the reperfusion normalized the IR-induced increases in the P-gp in the upper, middle, and lower intestine and CYP3A in the upper intestine. Additionally, Trolox administration prevented the IR-induced decrease in the oral availability of cyclosporine. The normalization of intestinal P-gp/CYP3A and blood AUC of cyclosporine by Trolox in IR rats suggests that the hepatic IR causes overexpression of intestinal P-gp and CYP3A through oxidative stress. The authors also showed that an increase in the intestinal CYP3A activity was at the transcriptional level, which they attributed to the increase in the bile acid levels in the intestine after hepatic IR. However, the IR-induced increase in the protein expression of intestinal P-gp was in the absence of changes in the mRNA levels of Mdr1a and Mdr1b, implying post-transcriptional upregulation of intestinal P-gp by oxidative stress and IR [98].

**Table III:** Effects of Liver Ischemia–Reperfusion Injury or Partial Hepatectomy on Drug Transporters in Remote Organs

Species	Intervention	Remote Organ	Transporter Change	Technique	Reference
Wistar Rats	Warm partial (70%) ischemia (60 min/reperfusion (12 h)	Intestine	P-gp⇑ (apical membrane/intestinal lumen)	WB/intestinal tissue, *in vivo* oral bioavailability of cyclosporine, and intestinal absorption of cyclosporine in upper parts of *in situ* closed loop intestinal model	[71, 98]
SD Rats	Warm partial (70%) ischemia (60 min)/reperfusion (0–48 h)	Kidneys	Mrp2⇑, Mrp4⇑ (apical membrane/brush border membrane/urine)	mRNA and WB/kidney tissue	[69]
			Mrp3⇑ (basolateral membrane/blood)	mRNA/kidney tissue	
Wistar Rats	Warm partial (70%) ischemia (60 min)/reperfusion (12 h)	Kidneys	rOCT2⇓ (basolateral membrane/blood)	Total, renal, and tubular secretion clearance of cimetidine	[99]
			rOCT2⇓, rOAT3⇓ (basolateral membrane/blood) rMATE1⇓ (apical membrane/brush border/urine)	WB/Kidney Tissue	
SD Rats	Warm partial (70%) ischemia (90 min)/reperfusion (24 h)	Brain	P-gp⇑ (apical membrane/blood)	WB/brain membrane fractions; *in vivo* brain uptake clearance of RH-123	[66]
ddY Mice	Partial (70%) hepatectomy (24 h after surgery)	Brain	Mdr1a⇔, Mdr1b⇔	mRNA/cerebral capillaries	[100]
			P-gp⇓ (apical membrane/blood)	Brain uptake of cyclosporine	
Wistar Rats	Partial (70%−75%) hepatectomy (1 and 7 days after surgery)	Intestine	Mrp2⇔ 1 and 7 days after surgery (apical membrane/intestinal lumen)	WB & immunofluorescence/jejunum	[95]

In addition to the small intestine, the remote effects of warm hepatic IR on transporters have also been reported for kidneys (Table III) [69, 99]. Tanaka *et al.* [69] reported that whereas warm 70% hepatic IR in rats decreased the mRNA and protein levels of Mrp2 in the liver (Table II), the expression of Mrp2 in the kidneys substantially increased (Table III). In the kidney tissue, the mRNA levels of Mrp2 significantly increased by 29% and 283% after 3 and 6 h of reperfusion, respectively. In comparison, the protein levels of Mrp2 were increased by 188%, 381%, and 490% after 6, 24, and 48 h of reperfusion, respectively [69]. The hepatic IR-induced increases in the protein levels of Mrp2 in the kidney were further confirmed by immunohistochemistry analysis, which showed significant expression of Mrp2 at the brush border membrane of the proximal tubules 24 h after hepatic IR [69]. Although the exact mechanisms were unclear, the authors proposed that the hepatic IR-induced increases in the serum concentrations of conjugated bilirubin and/or bile acids could upregulate renal Mrp2 via nuclear receptors, such as Nrf2. Nevertheless, an IR-induced increase in the Mrp2 levels in the kidneys may protect the kidneys from the harmful effects of liver IR injury by facilitating the excretion of toxic substances into the urine.

The remote effects of hepatic IR on kidney transporters and their implications for the pharmacokinetics of drugs were further reported by Okuda and colleagues [99]. These investigators showed that warm 70% ischemia (60 min)-reperfusion (12 h) in the liver caused 33%, 39%, and 35% reductions in the expression (protein) levels of the rat basolateral organic cation transporter 2 (rOCT2), luminal multidrug and toxin extrusion 1 (rMATE1), and basolateral organic anion transporter 3 (rOAT3), respectively. *In vivo* pharmacokinetics of cimetidine was also studied in the sham and IR animals after the IV administration of the drug, the results of which are summarized in Table IV. Cimetidine is a cationic drug, which is eliminated by both hepatic metabolism and renal excretion of the unchanged drug, with the latter pathway playing a more prominent role in its elimination. Hepatic IR injury in rats caused 22% and 37% reductions in the total (CL) and renal (CL_R_) clearance of cimetidine (Table IV). Whereas the glomerular filtration rate and clearance of cimetidine by renal filtration (CL_GFR_) were the same in both sham and IR animals, the tubular secretory clearance (CL_TS_) of cimetidine was reduced by 45% in the IR animals (Table IV). Because the tubular secretion of cimetidine is through renal organic cation transporters rOCT2 and rMATE1, the authors attributed the reductions in the tubular secretion and CL_R_ of cimetidine in the IR rats to the reductions in the protein levels of these transporters in these animals [99]. In addition to rOCT2 and rMATE1, the decrease in rOAT3 might have also contributed to the reduced renal excretion of cimetidine because the drug is also recognized by rOAT3. Interestingly, the administration of the antioxidant Trolox before the reperfusion abrogated the IR-induced declines in the protein levels of rOCT2, rMATE1, and rOAT3 and the clearance of cimetidine, suggesting a likely role for oxidative stress in the observed effects of hepatic IR on renal transporters [99].

**Table IV:** Total (CL), Renal (CL_R_), Filtration (CL_GFR_), and Tubular Secretory (CL_TS_) Clearance (mean ± SD) of Cimetidine in Sham and Hepatic IR Rats

	Sham	IR
CL, mL/min/kg	43.6 ± 3.4	34.2 ± 3.2^a^
CL_R_, mL/min/kg	24.6 ± 5.1	15.4 ± 1.4^b^
CL_GFR_, mL/min/kg	4.72 ± 0.18	4.49 ± 1.4
CL_TS,_ mL/min/kg	19.9 ± 5.0	11.0 ± 0.3^b^

Based on data reported by Ikemura *et al.* [99]

^a^*p* < 0.01 and ^b^, *p* < 0.05, compared with sham (unpaired *t*-test)

In a model of warm 70% ischemia (90 min)-reperfusion (24 h) injury in the rat liver, we [66] showed that, in addition to the liver, the P-gp protein levels in the remote organ brain were significantly increased by ~ 30% (Table III). The ~ 30% increase in the brain levels of P-gp was associated with ~ 30% decline in the brain uptake clearance of RH-123, a substrate for P-gp. The hepatic IR-induced upregulation of P-gp in the brain [66] is in agreement with the reported [71, 98] upregulation of the transporter in the intestine, discussed above.

In contrast to the hepatic IR-induced upregulation of brain P-gp, a study in mice [100] (Table III) reported a 15%−22% increase in the brain concentration of cyclosporine, a P-gp substrate, 24–72 h after two-thirds partial hepatectomy, which the authors attributed to a decrease in the brain function of P-gp as a result of partial hepatectomy. However, the mRNA levels of Mdr1a and Mdr1b remained unchanged 1–7 days after the surgery. A limitation of this study is that the brain concentrations of cyclosporine were not corrected for the plasma/blood concentrations of the marker to account for potential changes in the systemic exposure of cyclosporine because of hepatectomy. As we have reported before [101, 102], using the brain concentrations of drugs alone as a measure of changes in brain uptake without correcting for potential changes in their plasma/blood AUC may be misleading. Therefore, whether the increased brain concentration of cyclosporine in hepatectomized mice is due to an increase in its blood AUC or a decrease in the brain P-gp function remains to be determined.

### Effects of Liver Surgery on Drug Transporters in Humans

As stated in the above sections, the overwhelming majority of studies on the effects of liver surgery on drug transporters are conducted in rodents, particularly rats (Tables I–IV). Table V summarizes the few studies in the literature on the effects of liver surgery on drug transporters in humans. In agreement with the rodent studies showing the upregulation of P-gp in hepatic IR, Grude *et al.* [10] showed that P-gp was overexpressed after liver transplantation in humans. Flow cytometric analysis of peripheral blood mononuclear cells showed a ~ 30% increase in the P-gp expression on day 7 after transplantation, compared with the data before transplantation. The ~ 30% increase in P-gp on day 7 persisted and remained relatively stable on days 14, 21, 28, 90, and 180 days after the transplantation. Treatment of the patients with cyclosporine or tacrolimus did not significantly affect the extent of overexpression of P-gp post-transplantation.

**Table V:** Effects of Liver Surgery on Drug Transporters in Humans

Intervention	Outcome	Technique	Reference
Liver transplantation	P-gp⇑ on days 7 to 180 post-transplantation	Flow cytometry/peripheral blood mononuclear cells	[10]
Recipient with a second living-donor liver transplant (LDLT)	P-gp⇑ (compared with first LDLT)	mRNA and WB/enterocytes	[7]
	Reduced Bioavailability of cyclosporine	Blood cyclosporine levels	
Living-donor & deceased-donor liver transplantation	In 83% of recipients, the pattern of distribution of MRP2 changed from homogeneous canalicular staining (normal liver) to focal canalicular plus partial membranous staining of cytoplasm or only membranous (no canalicular) staining	MRP2 antibody staining of liver biopsy tissue	[103]
Liver Transplantation	BSEP⇔ at the end of cold preservation or 3 h after transplantation (compared with control livers) BSEP⇑ 1 week after transplantation (compared with control livers and liver grafts at the end of cold preservation or 3 h after transplantation)	mRNA/liver tissue	[104]
Partial Hepatectomy (Pringle Maneuver)	NTCP⇔, BSEP⇔, MRP2⇔ 30 min after reperfusion	mRNA/liver tissue biopsy before and after Pringle Maneuver	[105]

Another study [7] investigated the intestinal P-gp and CYP3A expression and oral immunosuppressive requirement in a patient who received a second LDLT after chronic rejection of the first graft. The investigators reported that the blood concentration: dose ratio of oral tacrolimus was similar to the average for other LDLT patients after the first LDLT. However, the patient required substantially higher than average oral doses of cyclosporine after the second LDLT. The average blood concentration: oral dose ratio of cyclosporine in the patient after the second LDLT was ~ 80% lower than the ratios in other LDLT patients in the same hospital. In contrast, the patient’s ratio after the IV administration of cyclosporine was similar to that in other patients. Comparing the average blood concentration: dose ratios after oral and IV administration of cyclosporine in this patient, the authors estimated that the oral bioavailability of cyclosporine in the patient was 6.6%, substantially lower than in other patients. The protein levels of P-gp in the upper jejunum enterocytes, determined by Western blot analysis, showed a significant increase on day 184 compared to day 0 after the second LDLT. The authors suggested that the decrease in the blood concentrations of cyclosporine after the second LDLT in this patient was due to an overexpression of intestinal P-gp.

In another case of agreement between the experimental models and humans, Yi *et al.* [103] reported changes in the cellular localization of liver MRP2 after liver transplantation in humans, which were similar to those reported after both cold [75, 77] and warm [42] hepatic IR injury in rats. The authors used immunohistochemical staining to investigate the cellular localization of MRP2 in the liver biopsies obtained within two months of transplantation from 19 LDLT patients and 22 DBD patients. For control, liver biopsies from 15 donors for the LDLT were stained. The control livers showed homogeneous MRP2 staining of the canalicular area without any staining in the cytoplasmic region. Whereas 17% of the graft livers showed homogenous MRP2 canalicular distribution, 83% of the grafts showed altered localization of cellular MRP2 with partial or complete relocalization of MRP2 from the canalicular membranes into the cytoplasm. The extent of redistribution of MRP2 from the canalicular membrane to the cytoplasm was positively correlated with the length of operation and post-surgery complications [103]. The similar cellular relocalization of MRP2 in liver transplant patients [103] and rats subjected to cold [75, 77] or warm [42] IR injury suggests that the observed phenomenon in humans is likely due to the IR injury of the graft during liver procurement, storage, and/or implantation.

In another human study [104], liver biopsies were obtained from 24 livers at the end of cold preservation and at 3 h and one week after the reperfusion of the transplanted graft, and the mRNA levels of BSEP were measured (Table V). Additionally, BSEP mRNA levels were determined in control livers. The authors reported that the BSEP mRNA levels at the end of cold preservation or 3 h after the reperfusion of the transplanted grafts were not significantly different from the control livers. However, the levels of BSEP mRNA one week after the operation were significantly higher than those for the control livers or liver grafts at the end of cold storage or 3 h of reperfusion (Table V). The increase in the BSEP mRNA one week after liver transplantation in humans is in agreement with a study [81] in rats that showed seven days after transplantation of liver grafts that were stored in cold for 1 or 12 h, the BSEP mRNA levels were increased. However, in the rat study, the mRNA levels were normal or declined on days 1, 3, and 5 post-transplantation (Table I). Whether the reported changes in the BSEP mRNA levels after liver transplantation in humans cause any changes in the protein levels or function of BSEP remains to be investigated.

A potential complicating factor in interpreting the effects of liver transplantation on the function of drug transporters and pharmacokinetics of drugs used in these patients is the donor and recipient genetic differences in drug transporters and metabolizing enzymes. After oral administration, the drug is first exposed to the recipient’s pharmacogenomics in the gastrointestinal tract, followed by exposure to the donor’s pharmacogenomics in the liver [106]. Indeed, it has been reported that genetic polymorphisms in the recipient ABCB1 (P-gp) and donor CYP3A significantly affect tacrolimus pharmacokinetics and dose requirement in the immediate post-transplant period [107, 108]. Additionally, the recipient’s ABCB1 genotype was significantly associated with the risk of chronic rejection after the administration of calcineurin inhibitors in liver transplant patients [109]. Whether the effects of liver surgery on drug transporters are dependent on the donor graft genotype and/or the recipient genotype remains to be elucidated.

The effects of the Pringle maneuver on the mRNA levels of NTCP, BSEP, and MRP2 were investigated in patients undergoing partial hepatectomy [105] (Table V). The investigators took two liver biopsies, one before the start of the resection and another 30 min after removing the clamp and the beginning of the reperfusion, in a procedure with an ischemic time of 43 ± 22 min (mean ± SD). The mRNA levels of the three studied transporters were similar before and 30 min after the Pringle maneuver, suggesting a lack of acute effects of the procedure on the transporters. However, the long-term effects of the Pringle maneuver or partial hepatectomy on these transporters were not investigated.

## Conclusions and Perspectives

Liver surgeries, such as liver transplantation and resection, are associated with a significant increase in the generation or release of many inflammatory mediators, such as ROS, pro-inflammatory cytokines, DAMPs, and adhesion molecules. The increased inflammatory mediators may alter the expression and function of drug transporters in the liver and remote organs, such as the intestines, kidneys, and brain. Most of the studies in this area have been conducted in animal models, with limited reports in humans. Animal studies show that the effects of IR injury and partial hepatectomy on drug transporters are complex and depend on many variables, such as the species, length and type of ischemia, reperfusion time, and the extent of liver resection. Therefore, the effect of liver surgeries on drug transporters is hard to predict in most cases. However, this review has revealed some trends and agreements between the more extensive animal studies and the limited human reports. A major finding is that both animal and human studies show a clear overexpression of P-gp in the liver and remote organs after warm (normothermic) hepatic IR injury or liver transplantation. The P-gp overexpression could have major clinical ramifications, particularly in patients with liver transplantation who receive P-gp substrates like immunosuppressants. Another observed trend is the relocalization of liver MRP-2/Mrp2 from the canalicular membranes to the cytoplasmic area, reducing the function of the transporter even in the absence of a change in its protein. Nevertheless, the clinical data in humans related to the effects of liver surgeries on these two and other drug transporters are scarce. Future studies should concentrate on the impact of liver surgeries on the time course of potential changes in the expression and/or function of drug transporters in humans.

## Abbreviations

ABC: ATP-binding cassette
ADP: Adenosine diphosphate
ATP: Adenosine triphosphate
AUC: Area under the plasma or blood concentration–time curve
BCRP/Bcrp: Breast cancer resistance protein
BSEP/Bsep: Bile salt export pump
CL: Total (systemic) clearance
CL_GFR_: Filtration clearance
CL_R_: Renal clearance
CL_TS_: Tubular secretory clearance
CYP3A: Cytochrome P450 3A
DAMP: Damage‐associated molecular pattern
DBD: Donation after brain death
DCD: Donation after cardiac death
FXR: Farnesoid X receptor
HIV: Human immunodeficiency virus
HMGB1: High mobility group box 1
IFN-γ: Interferon-gamma
IL-1β: Interleukin 1-beta
IL-12: Interleukin 12
IPRL: Isolated perfused rat liver
IR: Ischemia-reperfusion
IV: Intravenous
LDLT: Living donor liver transplantation
MDR/Mdr: Multidrug resistance protein
MFA: Mycophenolic acid
MRP/Mrp: Multidrug resistance-associated protein
Nrf2: Nuclear factor erythroid 2-related factor 2
NTCP/Ntcp: Sodium-taurocholate co-transporting polypeptide
OAT/Oat: Organic anion transporter
OATP/Oatp: Organic anion-transporting polypeptide
OCT/Oct: Organic cation transporter
P-gp: P-glycoprotein
PXR: Pregnane X receptor
RH-123: Rhodamine 123
RH-Glu: Rhodamine 123 glucuronide
rMATE: Rat multidrug and toxin extrusion type transporter
rOAT: Rat organic anion transporter
rOCT: Rat organic cation transporter
ROS: Reactive oxygen species
SLC: Solute carrier
TNF-α: Tumor necrosis factor-alpha
WB: Western blot
